# Sink-oriented Dynamic Location Service Protocol for Mobile Sinks with an Energy Efficient Grid-Based Approach

**DOI:** 10.3390/s90301433

**Published:** 2009-03-03

**Authors:** Hyeonjae Jeon, Kwangjin Park, Dae-Joon Hwang, Hyunseung Choo

**Affiliations:** 1 School of Information and Communication Engineering, Sungkyunkwan University, 300 Cheoncheon-dong, Suwon Gyeonggi-do 440-746, South Korea; E-Mails: jeonnow@skku.edu; kjpark@wonkwang.ac.kr; djhwang@skku.edu; 2 Division of Electrical Electronic and Information Engineering, Wonkwang University, 344-2 Shinyong-dong, Iksan Chonbuk 570-749, South Korea

**Keywords:** Wireless Sensor Networks, Data Dissemination Protocol, Location-based Routing, Mobile Sink, Energy Efficiency, Lifetime

## Abstract

Sensor nodes transmit the sensed information to the sink through wireless sensor networks (WSNs). They have limited power, computational capacities and memory. Portable wireless devices are increasing in popularity. Mechanisms that allow information to be efficiently obtained through mobile WSNs are of significant interest. However, a mobile sink introduces many challenges to data dissemination in large WSNs. For example, it is important to efficiently identify the locations of mobile sinks and disseminate information from multi-source nodes to the multi-mobile sinks. In particular, a stationary dissemination path may no longer be effective in mobile sink applications, due to sink mobility. In this paper, we propose a Sink-oriented Dynamic Location Service (SDLS) approach to handle sink mobility. In SDLS, we propose an Eight-Direction Anchor (EDA) system that acts as a location service server. EDA prevents intensive energy consumption at the border sensor nodes and thus provides energy balancing to all the sensor nodes. Then we propose a Location-based Shortest Relay (LSR) that efficiently forwards (or relays) data from a source node to a sink with minimal delay path. Our results demonstrate that SDLS not only provides an efficient and scalable location service, but also reduces the average data communication overhead in scenarios with multiple and moving sinks and sources.

## 1. Introduction

Recent developments of wireless communication and Micro Electro Mechanical Systems (MEMS) technologies have made possible the deployment of large-scale Wireless Sensor Networks (WSNs) [1]. WSNs can be used for a wide range of applications in the scientific, commercial, medical, and military battlefield, industry control, traffic control, and ambient conditions detection areas [1–3].

Typical WSNs are composed of a large number of sensor nodes which transmit the sensed information to the sink. Since a sensor node is constrained by a device with limited power supply, recharging sensor nodes is often infeasible. Energy efficient algorithms are one of the most important challenges in large-scale WSNs, since sensor nodes have restricted energy. For example, WSNs may not fulfill their function normally if some sensor nodes fail due to insufficient power. Therefore, it is important to reduce the energy consumption of sensor nodes and to maximize the lifetime of the entire network [4–10].

Most existing studies on data dissemination protocols in WSNs are designed for stationary sensor nodes and sinks [4–10]. Although these protocols have not been designed to provide sink mobility, the mobile sink is used by current applications such as mobile phones and PDAs carried by itinerant users. In the mobile sink environment, the connection of the last hop to the mobile sink will be broken if it does not promptly reflect the updating of the relay path from the mobile sink. Many researchers have recently studied the problem of scalable and efficient data dissemination in large-scale WSNs from multiple sources to multiple mobile sinks [11–24].

**Figure 1.**
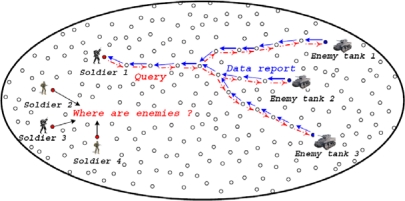
A sensor network example. Soldiers use the sensor network to detect tank locations.

Let us consider a monitoring application as an example. Assume that soldiers collect the information on the enemy's emergence from WSNs deployed in a battlefield (see Figure 1 for an example). A sink is a soldier that collects the sensed information from WSNs. WSNs may not execute their function normally if some sensor nodes fail due to energy exhaustion. Therefore, it is important to reduce the energy consumed by the energy-limited sensor nodes that continuously provide the data reports to soldiers in a battlefield. This scenario motivates us in this paper.

In this paper, we propose a Sink-oriented Dynamic Location Service (SDLS) protocol to handle sink mobility. SDLS provides a complete solution to support both sink mobility and energy conservation. To the best of our knowledge, this is the first work on a Sink-oriented (user-oriented) data dissemination approach for supporting general data delivery to multiple, mobile sinks in WSNs. Our contribution is as follows:

1. We propose a global-grid structure to avoid repetitively constructing an individual grid structure for each source node. The global grid structure helps reduce overall energy consumption. A sink can use the global grid structure to identify the location of the source node whenever it wants to do so.
2. We propose an Eight-Direction Anchor (EDA) system that acts as a location service server. EDA prevents intensive energy consumption at the border sensor nodes; consequently, the energy consumed is well balanced for all the sensor nodes.
3. We propose a Location-based Shortest Relay (LSR) that efficiently forwards (or relays) data from the source node to the sink with minimal path delay. Our protocol does not need a global grid structure anymore, once a sink identifies the location information of the source node by using global grid structure.
4. We evaluate performance on a JAVA implementation of SDLS. We demonstrate that the proposed protocol not only reduces communication costs, but also balances energy consumption.

The remainder of the paper is organized as follows. Section 2 introduces related work supporting mobile sinks. Section 3 details the SDLS protocol and Section 4 evaluates performance. Finally, Section 5 concludes the paper.

## 2. Related Work

### 2.1. Grid Structure Data Forwarding Approach with Mobile Sink

Several protocols have been proposed to implement query and data dissemination architectures in WSNs [8–10]. Most of the aforementioned studies on data dissemination protocols still assume both a single sink and knowledge of the sink's location, but mobile sink environments are used by many current applications such as mobile phones and PDAs, so many researchers have recently studied the problem of supporting sink mobility [11–24].

EEDD [14] has proposed an Energy-Efficient Data-Dissemination protocol to solve both the target and inquirer mobility problem. It has adopted a virtual grid-based two-level design to schedule the active sensor nodes. GGR [15] is a proposed Geographic Grid Routing protocol to provide a multi-path approach at all stages of communication. It focuses on the reliability aspects of the routing protocol.

Perhaps the most similar protocol to SDLS is the Two-Tier Data Dissemination (TTDD) protocol [16], which is source oriented to provide the location information of the source node. TTDD is a well-known grid-based routing protocol to provide query and data dissemination for multiple mobile sinks. Upon detection of an event, the source node proactively creates a virtual grid structure throughout the sensor field with dissemination nodes located at the grid cross points. TTDD operates in a two-tier hierarchical manner to support the mobile sink’s routing. First, the lower tier is within the cell of the sink’s current location. When the sink wants to obtain the sensed data, it floods its query within the lower tier until it reaches the closest dissemination node, called the immediate dissemination node. Second, the query message is then forwarded through the higher tier, which is made up of either the source node or a dissemination node from the sink’s cell to the source’s cell. The dissemination node requests a data download to the source node along the reverse grid path with the data announcement. Finally, once the source node receives the query message, packets are forwarded from the source to the immediate dissemination node along grid horizontal and vertical axes. Requested data will flow down in the reverse direction to the sink. Thereafter, the requested data from the source node is forwarded by trajectory forwarding [16]. TTDD solves the sink mobility problem using a grid structure. However, if the number of source nodes is increased, data dissemination point management can considerably increase the communication and storage overhead of the system, due to the grid structures of data dissemination points from each source node [23]. Therefore, an efficient protocol with multiple source nodes is necessary [12–15,20–24].

### 2.2. Location Service for a Location-based Approach with Mobile Sinks

Several location-based routing approaches have been considered to address effectively the location tracking problem. However, most of the existing studies on location service protocols were designed for MANETs [17, 18]. Directly employing them in WSNs is ineffective. Location-based routing has been considered to address the issue of providing locations as an effective routing approach in WSNs [19–24]. If the sensor node's location information is found, most applications can use this information to make routing decisions. Greedy Perimeter Stateless Routing (GPSR) [26] is a geographic routing system for multi-hop wireless networks. If a source node can identify a destination node's location, GPSR can forward a packet to the nearest neighbor node in order to reach the destination node.

A locator [20] has been proposed as a data dissemination architecture to support mobile sinks. Once a sensor node wants to report its sensed data to sinks, the source can obtain the current sinks' locations from the locators. These locators are selected for the sensor fields using a deterministic geographic hash function and are replicated uniformly. Railroad [21] has proposed a data dissemination architecture by proactively exploiting a virtual infrastructure called rail. This rail is placed in the middle of the sensor field where all the metadata from the event data are stored. The rail acts as a meeting area for the events and queries. Once a source node detects an event, the data remain stored locally and only relevant metadata are sent to the rail. Then, the sink can retrieve metadata with the queries circulating around the rail. When corresponding metadata is found, the source node of the data transmits the corresponding data to the sink node that has issued the query. Hierarchical Location Service [22] has been proposed as a scalable hierarchical location service in which the location updating structure for the mobile sink is hierarchically built, which can reduce the updating cost. Further, the overall updating cost in WSNs with multiple sinks is greatly reduced by enforcing Voronoi scoping in which each mobile sink only updates its location to the location servers closest to it.

Perhaps the most similar protocol to SDLS is the Anchor Location Service (ALS) protocol [23]. ALS is a grid-based protocol that provides the location information of the sink. ALS constructs a single global grid structure, assigning the sensors nearest the grid points as grid nodes. The sink selects the nearest grid node as its sink agent to distribute its location information. At the beginning of the process, the sink agent sends out four anchor setup messages in four straight orthogonal directions (North, South, East, and West) and all the grid nodes that lie along the routing path are recruited as anchors. Once an event occurs, the source node will register with the nearest grid node, known as the source agent. The source agent will then send four location query packets to find the location of the sink agent. It then receives the location of the sink agent. The source node finally sends the data packets to the sink agent using GPSR protocol. However, it is difficult to identify the location of a moving sink. The following problem occurs when the sink constructs an anchor system. First, the source node cannot identify the location of the sink if the sink does not construct an anchor system. Second, the sink has to wait until the sensor node detects the event. Third, when the sink has high mobility, the sink agent has to relay the data from the source node to the sink. Thus, the distance between the source node and the sink may increase significantly when a detour occurs. ALS also suffers from concentrated energy consumption because it always uses border nodes when it provides the sink location. Therefore, it is necessary to propose an energy efficient protocol to balance sensor node energy consumption.

The Sink Location Service (SLS) [24] informs the source node about the sink location to discover sink locations with low overhead. Each senor node gets four anchor locations during network initialization phase. A source node and a sink send sink location announcement and query messages along two paths respectively by geographic routing. The sensor node located on the crossing point of the sink location announcement path and the sink location query path informs the source node of the sink location, then the source sends the data packet to the sink by geographic routing. No flooding or global grid structure is needed for sink location announcement or query. Although SLS provides sink locations to minimize the overhead incurred for the provision of source nodes with the location of sinks, it does not solve forwarding data from a source node to a mobile sink. This process decreases the resilience of the protocol. The main reason for this is if one query message fails, there is no other query message to retrieve sink location information. In addition, no sensor node might be located on the crossing point of these two paths. In this case, the source node cannot find the location information of the sink.

## 3. Sink-oriented Dynamic Location Service

In this section, we describe in detail the SDLS protocol. SDLS works under the following basic network assumptions:

1. Sensor nodes are placed in two-dimensional square field where they are randomly deployed with high density.
2. Sensor nodes communicate with each other using single-hop communications through short-range radios. Long-range data delivery is accomplished forwarding data across multiple-hops.
3. Each sensor node knows its own location, as well as the location of its 1-hop neighbor nodes using localization algorithms [25].
4. Upon detecting an event of interest, a source node immediately reports its location information using the Eight-Direction Anchor (EDA) system.
5. One or more mobile sinks moves randomly in the deployment field. Sinks (users) query the network to collect the location of source nodes. Then, sinks wait until they receive the location of source nodes. After getting the location of the source node, the sink can dynamically send the data request query to the source node with consideration of source node’s location and direction of sink’s movement. Therefore, we named our proposed scheme as Sink-oriented Dynamic Location Service (SDLS) protocol.

### 3.1. Motivation

When a sink sets on static location, geographic routing can be used successfully, i.e. sending the packets to the closest node to the destination. As for the static sink, it only needs to broadcast once to the whole network at the initial stage of the network operation, which would not result in significant energy dissipation. If a sink moves quickly to be out of last hop node’s transmission range, geographic routing can’t be executed correctly without a mobile sink’s location updates because the sink can’t receive last hop forwarding without any additional reactions. For solving this problem, the mobile sink can periodically flood its location to all sensors but it is not efficient and scalable for large sensor networks and each time the sink changes its location in the network. Due to high packet overhead of flooding, when these nodes use up their energy, no more data can be transmitted back to the sink. Thus, sinks should update limited number of sensors own current location instead of flooding to all sensors. Therefore, the mobile sink only needs to broadcast to a subset of nodes in the network each time when it arrives at a new site, thus saving much energy.

**Figure 2.**
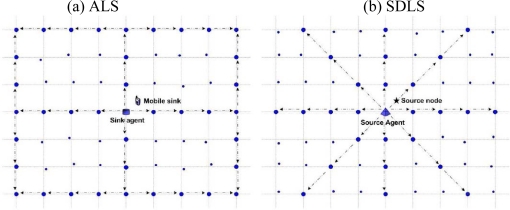
Selection of location service servers.

In a general way, a source node that has detected an event of interest tries to send its data to the sink. Therefore, the mobile sink broadcasts its location information over the subset of nodes in the network [see Figure 2(a)]. However, when the sink moves out of a destination node, it has to rebroadcast its location information over the network to a new destination node. We argue that this repeated network-wide broadcasting brings extra high energy loss which could impair the lifetime gains from sink mobility, especially in large-scale WSNs.

To address this problem, we provide source node’s location in the WSNs [see Figure 2(b)]. A significant difference between our proposed scheme and the traditional mobile sink supporting scheme is that the source node provides its location service server. Therefore, it is necessary to propose an energy efficient location service protocol to balance sensor node energy consumption.

In this paper, we propose a data dissemination model called EDA (Eight-Direction Anchor) for geographic. Sinks should be able to acquire current source node’s location with bounded time and overhead. EDAs are location server sensors that track source node’s position. Figure 2 illustrates our motivation for proposing the location service servers. As shown in Figure 2(a), selection of location service servers will be established using six lines of grid nodes, three lines of grid nodes traversing the network vertically and three lines of grid nodes traversing the network horizontally. But from Figure 2(a), EDA can reduce the traversing path from 6 to (2+2 $\sqrt{2}$) compared with ALS. EDA prevents intensive energy consumption at the border sensor nodes; consequently, the energy consumed is well balanced for all the sensor nodes.

**Figure 3.**
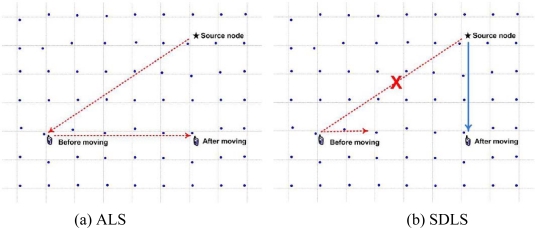
Selection of shortest path relay.

After the source node gets the sink’s location, the source node should be able to transfer the sensed data to shortest path. Figure 3 illustrates our motivation for proposing the shortest path relay.

When the sink has mobility, the sink agent has to relay the data from the source node to the sink. Then, this occasions a detour case (see Figure 3(a)). Therefore, it is necessary to propose an energy efficient shortest path relay. In our proposed algorithm, once the sink is aware of the source node’s location, the sink can directly request the data forwarding from the source node [see Figure 3(b)]. It can efficiently forward data from a source node to a sink with minimal delay path. Therefore the sink can quickly get source node’s data.

### 3.2. Global Grid Construction

SDLS constructs a shared global grid structure once all the sensor nodes are deployed. Global grid construction can be enabled when all sensor nodes obtain their locations using a local positioning system and maintain their one-hop neighbors’ geographic locations in their tables. Specifically, the grid is set up in parallel with the *X*-axis and *Y*-axis of the positioning system. The positive directions of the *X*- and *Y*-axes of the predefined positioning space are pointing to the east and north, respectively. The baseline coordinate (*Xbase*, *Ybase*) of a pre-defined positioning system is set in the mission messages or hard coded in each sensor node. The all sensor field is divided into small virtual grids. The basic idea of the grid-based approach is to establish a grid using the intersection points called grid points. The distance to the grid point is the cell size and is determined by the user such that grid points are not within transmission range. The sensor field is divided into square-shaped grids of user-defined size α. Each cell is an α × α square. Based on the global grid, the virtual coordinate (*Xp*, *Yp*) is determined to be grid point, the nearest node to the grid point is called the “grid node”, and the grid nodes that take the role of the location server for a specific source node are named “anchors”. The grid points are determined using the baseline coordinate (Xbase, Ybase) as follows:

(1) {Xp=X base+i*α,Yp=Y base+j*α};{i,j=±0,±1,…}

The first grid point sends a grid node announcement message to each of the four adjacent grid points, using simple geographic forwarding through intermediate nodes. As a result, the node that is geographically closest to each of the grid points becomes the grid node. Finally, several nodes are selected as grid nodes to cover all grid points across the original network. Every grid node will be in charge of a location server and will maintain the location information of the source node in its cache. After selection, the grid nodes will announce their role to the adjacent grid nodes. Then, neighboring grid nodes send notification messages to each other to establish a neighbor grid node table. The main objective of global grid is to deliver location information of the source nodes to the multiple mobile sinks. In the case of multiple mobile sinks, it is difficult to keep tracking the location of mobile sinks, since links between the source node and the sink may be easily broken. In our protocol, once the sink obtains the location of the source node using the global grid structure, the sink can directly send a query to the source node. This will be explained in Section 3.5.

### 3.3. Eight-Direction Anchor System

Although some location services have been proposed to address location tracking, they suffer from inefficient energy consumption or their specifications are not detailed in. Especially, ALS [23] suffers from concentrated energy consumption, as it always uses border nodes, when it provides the sink location. Though SLS [24] generates lower overhead than existing protocols such as flooding, TTDD, and ALS, they do not consider both location response time and resilience in case one query message fails. Therefore, we propose the Eight-Direction Anchor (EDA) system to solve these problems. EDA is built using the following procedures.

**Figure 4.**
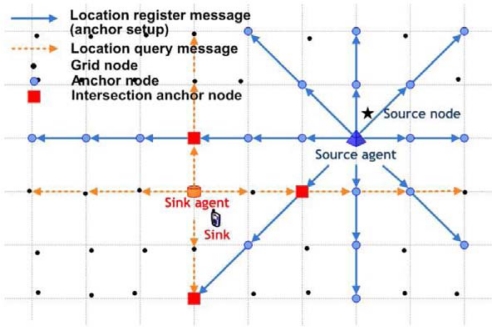
The anchor selection and the query and data dissemination processes.

1. Once an event occurs, a source node selects the nearest grid node as its source agent (see Figure 4, source node and selected source agent) and distributes the location information of the source node.
2. The source agent broadcasts the location of the source node in eight directions (East, West, South, North, Northeast, Northwest, Southeast, and Southwest) through anchor setup messages.
3. The anchor setup messages are relayed by intermediate sensor nodes between two neighboring grid nodes and the recruited anchors store a copy of the location of the source node.

These anchor setup messages are generated by a geographical routing protocol. That is, the source agent distributes the location information of the source node to its eight-direction neighboring grid nodes until the message reaches the network border. EDA can prevent intensive energy consumption at the border sensor nodes and considers location response time and success rate to obtain location information. Algorithm 1 explains how to make an Eight-Direction Anchor system at source nodes.

**Algorithm 1.** Pseudo-code to make an Eight-Direction Anchor system at source nodes.

**Procedure:**
01: initially startNode, middleNode, endNode = 0;
02: source node finds the nearest grid node as source agent
/* source node floods a query within a local area about a cell size. */
03: startNode = source agent;
04: **for** (source agent’s each neighbor grid node) /* to eight-direction */
05: endNode = neighbor grid node;
06: **while** (endNode != border node)
07: middleNode = **forward** (startNode, endNode);
/* finding the nearest neighbor node to reach the destination node */
08: **if** (middleNode == grid node)
09: **saveLocationServer** (source node’s location information);
10: **end if**
11: startNode = middleNode;
12: **if** (startNode == endNode)
13: endNode = startNode.neighbor grid node;
/* i.e.) if the message is from the east, startNode’s neighbor grid node is same direction. */
14: **end if**
15: **end while**
16: **end for**

### 3.4. Query and Data Dissemination

A sink queries the network to collect the location of source nodes. Query and data dissemination are achieved using the following procedures. After creating the anchor through the anchor nodes selection stage, the sink can identify the location of the source node by sending a query when the sink wants to receive events. To do this;

1. The sink will register with the nearest grid node, known as the sink agent (see Figure 4, sink and selected sink agent).
2. The sink agent sends four location query packets (East, West, South, and North) to find the location of the source node.
3. The sink can obtain the location of source nodes from the location response at intersecting anchor nodes (Figure 4).

Furthermore, once the sink agent obtains the source node’s location information, it does not obtain it again. If one query message fails, the other two queries will serve as backups to retrieve the source node’s location information. This also shortens the query response time, because the sink will utilize the first response.

Once the sink agent receives the location information of the source node, the sink agent forwards it to the sink. The sink then finally can communicate with the source node using the GPSR protocol.

Since our protocol provides the location of the source nodes, instead of the location of the mobile sinks, the anchor system, which is the location server, can maintain the location information of the source nodes, although the sinks move fast and continuously. Algorithm 2 explains how to find location of source nodes at sinks.

**Algorithm 2.** Pseudo-code to find location of source nodes at sinks.

**Procedure:**
01: initially startNode, middleNode, endNode = 0;
02: sink finds the nearest grid node as sink agent
/* sink floods a query within a local area about a cell size. */
03: startNode = sink agent;
04: **for** (sink agent’s each neighbor grid node) /* to four-direction */
05: endNode = neighbor grid node;
06: **while** (endNode != border node)
07: middleNode = forward (startNode, endNode);
/* finding the nearest neighbor node to reach the destination node */
08: **if** (middleNode == grid node)
09: **if** (exist a new source node’s location)
10: **getLocation** (sink agent); /* reply to the sink */
11: **end if**
12: **end if**
13: startNode = middleNode;
14: **if** (startNode == endNode)
15: endNode = startNode.neighbor grid node;
/* i.e.) if the message is from the east, startNode’s neighbor grid node is same direction. */
16: **end if**
17: **end while**
18: **end for**

### 3.5. Sink Mobility and Data Forwarding Maintenance

In this section, we propose a Location-based Shortest Relay (LSR) protocol to provide shortest path from a sink and a source node in mobile sink environments. In SDLS, once the sink obtains the location of the source node from the sink agent, the sink can directly send a query to the source node. Our protocol does not need to use a global grid structure to support a mobile sink. The sink selects the nearest sensor node as its Primary Agent (PA) to do so.

After the source node receives the query from the sink, it sends data to the PA. Thereafter, the source node can continuously send the data to the sink, once the data stream starts flowing. We now introduce the following efficient rules to support sink’s mobility. The algorithm 3 explains how to maintain sink mobility and data forwarding.

**Algorithm 3.** Pseudo-code to maintain sink mobility and data forwarding.

**Procedure:**
01: sink finds the nearest sensor node as sink primary agent
02: **if** (source node receives query message from sink)
03: source node sends data to sink
04: **end if**
05: **if** (source node continuously sends data to sink)
06: **if** (sink is automatic operation)
07: **go to**algorithm 3-1
08: **end if**
09: **if** (sink is manual operation)
10: **go to**algorithm 3-2
11: **end if**
12: **end if**

#### 3.5.1. Automatic Operation

The first rule is an automatic operation. When the sink moves out of the range of its current PA, within a predefined distance (e.g., a cell size), it selects another neighboring node as its Immediate Agent (IA) and sends the location of the IA to its PA. The sink continuously updates its instant location as its New Immediate Agent (NIA) so that future data are forwarded to the NIA. This process can be done by broadcasting a solicit message from the sink and then choosing the node that replies with the strongest signal-to-noise ratio. When the sink moves outside a predefined distance from its PA, it selects a New Primary Agent (NPA). If it only considers allowing the chain between the PA and the NPA as in ALS, the following quandary occurs. Even though the sink moves toward the source node, the sensed data from the source node takes a detour. This causes large energy consumption with a long data delivery when the source node repeatedly sends the data to the sink. In the SDLS, the previous distance from the source node to the last IA of the sink is compared with the current distance from the source node to the sink’s NPA. If the NPA is less than the threshold (i.e., half of previous distance), the NPA is updated through the Old Primary Agent (OPA). Until the source node obtains the location of the NPA, the OPA can forward the data continuously to the mobile sink. In particular, when the amount of data increases, the data forwarding cost will significantly increase. The following algorithm 3-1 summaries the first rule.

**Algorithm 3-1.** Pseudo-code to automatic operation.

**Input**: distance between source node and NPA
**Output**: shortest path in automatic operation
**Parameter:**
PA: Primary Agent, NPA: New Primary Agent
IA: Immediate Agent, NIA: New Immediate Agent
*l*1: distance between sink and PA (NPA)
*l*2: predefined distance (i.e. half of cell size)
*l*3: distance between PA and IA (NIA)
*l*4: predefined distance (i.e. three times of cell size)
*l*5: distance between source node and NPA via old PA
*l*6: predefined distance (i.e. two times of distance between source node and NPA)
**Procedure:**
01: initially startFlag = true;
02: **do**
03: **if** (startFlag)
04: **if** (*l*1 *> l*2)
05: find IA and send IA’s location information to PA
06: startFlag = false;
07: **end if**
08: **end if**
09: **else**
10: **if** (*l*1 *> l*2)
11: **if** (*l*3 *> l*4)
12: find NPA
13: **if** (*l*5 *> l*6)
14: **update** (NPA) /* update to source node */
15: **end if**
16: **end if**
17: **end if**
18: **else**
19: find NIA and send NIA’s location information to previous
20: **while** (sink has mobility)

#### 3.5.2. Manual Operation

**Figure 5.**
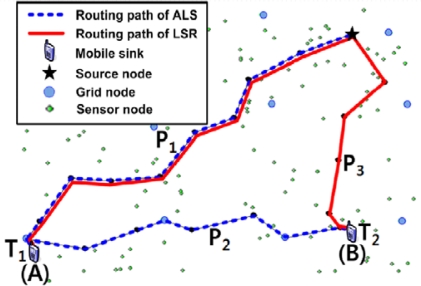
An example of the comparison of ALS and LSR to support sink mobility and data forwarding (extracted result from the simulator). Routing path of ALS and LSR = *P*_1_ (same) on point *A*, Routing path of ALS = *P*_1_ + *P*_2_ and LSR = *P*_3_ on point *B*.

The second rule is a manual operation. At that point, if the sink wants to move far-away, the sink informs its movement to the PA. Then, the PA notifies the source node to stop sending data. Nevertheless, the source node will continuously store the flowing data in its cache to prepare to resend the data. If the sink wants to renew the data flow, it sends the query through the new PA. The source node sends the data including the stored data in its cache. This approach is possible because the sink can quickly obtain the location of the source node whenever it wants. Let us consider an example in Figure 5. Assume that a mobile sink is located at points A, at time T1, and B, at time T2. The routing path for ALS and LSR at T1 is the same as P1. However, at T2, the routing path for ALS and LSR is P1 + P2 and P3, respectively. This is due to the LSR generating a new path based on the distance between the location of the source node with the current location of the sink and the last registered PA. Conversely, ALS simply forwards data without considering the distance between the location of the source node and the sink. The following algorithm 3-2 summaries the second rule.

**Algorithm 3-2.** Pseudo-code to manual operation.

**Input**: estimated distance between source node and sink
**Output**: shortest path in manual operation
**Parameter:**
PA: Primary Agent, NPA: New Primary Agent
**Procedure:**
01: **if** sink wants to move far-away from PA /* close to source node */
02: sink sends stop message to source node
03: **if** source node receives stop message
04: source node saves data in its cache
05: **end if**
06: **end if**
07: **if** sink wants to renew data
08: sink finds NPA and sends query message to source node
09: **if** source node receives query message
10: source node sends data including stored data
11: **end if**
12: **end if**

## 4. Performance Evaluation

In this section, we compare SDLS with TTDD, ALS, and SLS in terms of location service and data communication. We implement SDLS using JAVA to evaluate its performance. The simulation was implemented with the Java SE Development Kit (JDK) version 6. It is highly object oriented and helps flexibility and portability. We have obtained the same simulation result shown in the related paper with the simulator developed by authors. Additionally, we have various simulation results within following parameters. The default simulation setting has 200 stationary sensor nodes uniformly distributed in a two dimensional 1,000 x 1,000 m^2^ field. Some of the 200 sensor nodes are chosen as source nodes. Each simulation runs 100 times. That is, it is based on observations of 100 random sink and source node deployments. The radio transmission range is set to 200 m and the cell size is set to 200 m. We use the following energy model [27]:

$$\begin{array}{l} E_{\mathrm{tx}}=\alpha_{11}+\alpha_{2}\times d^{2}\\ E_{\mathrm{rx}}=\alpha_{12} \end{array}$$

where, E*_tx_* and E*_rx_* denote the energy consumed for transmitting and receiving a bit over distance d, respectively; α_11_, the energy/bit consumed by the transmitter electronics; α_2_, the energy dissipated in the transmit op-amp; and α_12_, the energy/bit consumed by the receiver electronics. The energy model has values of α_11_; α _12_ = 80 nJ/bit and α_2_ = 100 pJ/bit/m^2^. The control message making the anchor system and location query are each 36 bytes, while the data take up 64 bytes. We assume that the sink mobility pattern follows the widely-used random way-point mobility model [28]. The main parameters are listed in Table 1.

### 4.1. Average Energy Consumption for Location Service

**Figure 6.**
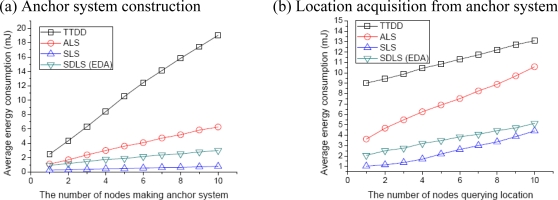
Average energy consumption for location service.

First, we compare the average energy consumption for location service. The location service is defined as receiving the location of all source nodes available in the network. Especially in SLS, if there is no node located on the crossing point of the two paths or one query message of the source node fails, the location service will continue until receiving all the location information of sinks. As the overhead to construct the grid (SDLS, ALS) or anchor nodes selection (SLS) is one time only, we do not include the results. Figure 6(a) shows a graph of each protocol’s average energy consumption versus the number of nodes making the anchor system from 1 to 10, incrementing by 1. While TTDD consumes more energy than the other protocols, SLS performs better than ALS and SDLS, since TTDD constructs grid structures (anchor system) for each source node and both ALS and SDLS are quite complex compared to SLS. ALS always uses the border nodes as its anchor system, so energy consumption is concentrated around border nodes. Conversely, SDLS balances across nodes in the entire grid. Therefore, it prevents high energy consumption of specific nodes. When we consider the energy consumption for making an anchor system, SLS provides the smallest energy consumption. However, this method will be disadvantaged in another evaluation.

Figure 6(b) shows the average energy consumption when nodes send a location query to find nodes to make an anchor system. Here, we consider that there are four nodes making the anchor system. TTDD requires more energy than the other protocols to make an anchor system; we can see that TTDD consumes more energy than the other protocols. TTDD also has to find the location of source nodes per each source node. However, both SDLS and ALS find node locations using global grid nodes with low energy consumption. The anchor system of ALS concentrates location information on border nodes. Thus, it frequently reaches the border nodes to obtain location information. Therefore, SDLS can obtain location information faster than ALS. As shown in the figure, we can see that ALS’s energy consumption increases faster than for TTDD. In TTDD, a sink finds four fixed source nodes. If the sink must find more source nodes, the protocol will consume more energy. As in forming the anchor system, SLS has the least energy consumption. However, the energy consumption of SLS increases gradually with the number of nodes querying location if no the sensor node is located on the crossing point or one query message fails.

### 4.2. Location Response Time

**Figure 7.**
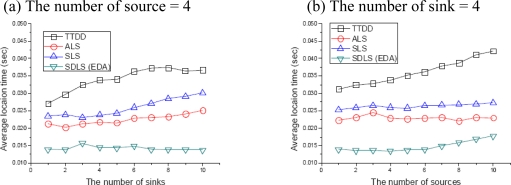
Location response time from anchor system.

In order to evaluate the average location response time of multiple sinks, we fixed the α = 200 m and varied the number of sinks from 1 to 10 in steps of 1. In ALS and SLS, from Figure 7(a), it can be seen that the location response time slightly increases with the number of sinks. This is because when multiple sinks are deployed in the network, location query messages always have to travel until they find all sink’s location information. ALS shortens the location response time than SLS, because ALS utilizes the first response among the other responses. However, because SDLS constructs anchor system by source node, the location response time remains fairly constant regardless of the number of sinks in the network. Although TTDD gets also fixed source nodes, it takes much time because TTDD constructs grid structures for each source node.

In order to evaluate the average location response time of multiple sources, we fixed the α = 200 m and varied the number of sources from 1 to 10 in steps of 1. In SDLS, from Figure 7(b), it can be seen that the location time slightly increases with the number of source. This is because when multiple sources are deployed in the network, location query messages always have to travel until they find all source’s location information. However, until source nodes are 10, SDLS is less time than ALS to get location information. TTDD is also same that the location time increases with the number of source nodes.

### 4.3. Remaining Energy Power of the Sensor Nodes for Data Communication

**Figure 8.**
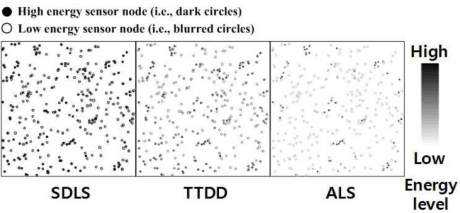
Remaining energy power of the sensor nodes for data communication.

To compare the impact of moving sinks on the location, we report the data communication overhead of SDLS, TTDD, and ALS except SLS, because SLS does not mention supporting mobile sinks after the location service. Figure 8 shows the remaining energy power of the sensor nodes for data communication. For the experiment, we assume the following parameter settings: number of sinks and source nodes = 30, number of sensor nodes = 400, size of the sensor field = 600 × 600 m^2^, transmission radius = 50 m, initial energy = 0.1 J, sink speed = 10 m/s, and the source node generates data packets 30 times. The data communication overhead is the highest with ALS. TTDD consumes less overhead than ALS, because TTDD can find a new path by switching to a new grid node. However, this is not the shortest path. Therefore, SDLS provides the best performance using the LSR protocol.

### 4.4. Average Data Delivery Ratio

**Figure 9.**
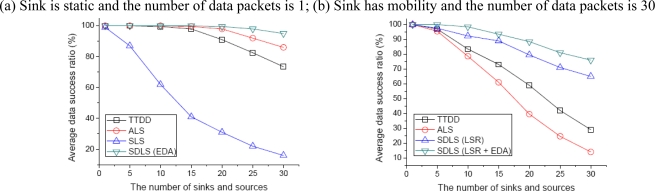
Average data success ratio.

Figure 9 depicts the average data success ratio, as the number of sinks and source nodes increases from 0 to 30 in increments of 5. From Figure 9(a), we assume that the sink is static and the source generates data packets only once with an initial energy of 0.1J. As shown in the figure, SLS has the lowest data success ratio. Here we can see that SLS has difficulty obtaining location information regardless of mobile sinks. Except for SLS, as shown in the figure, the average data success ratio decreases when the number of sinks and source nodes is over 15. However, SDLS still has a high success ratio, even when the number of sinks and source nodes is 30. From Figure 9(b), we assume that the sink speed is 10 m/s and the source node is generated 30 times with an initial energy of 1J. ALS has the worst data success ratio, as ALS has to continuously relay data, while SDLS provides the shortest path through the LSR for data transmission.

### 4.5. Network Lifetime

**Figure 10.**
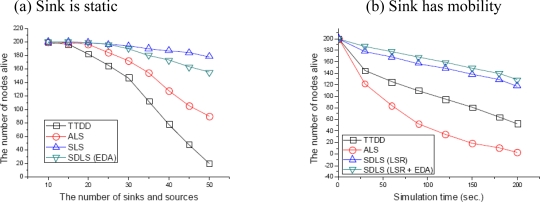
Network lifetime.

In Figure 10 (a), we simulated the network lifetime of each scheme according to the number of sinks and sources with an initial energy of 0.1 J. As shown in this figure, as the number of sinks and sources increases, network lifetime of all schemes decreases. Even though SLS has the highest network lifetime of all analyzed schemes, SLS has the lowest data success ratio as shown before. TTDD has the worst network lifetime because TTDD reselects a disseminate node for each source node whenever an event is detected. SDLS still has a high network lifetime even when the number of sinks and source nodes is 50.

Figure 10 (b) depicts the network lifetime according to the simulation time from 0 to 200 seconds. Sink speed is 10 m/s and the number of sinks and source nodes is 15, with an initial energy of 1J. Since SDLS distributes data over the entire network by considering residual energy, the network lifetime could be significantly prolonged compared to the other protocols. The network lifetime for both SDLS (LSR) and SDLS (LSR + EDA) is longer than other protocols.

### 4.6. Average Data Communication Overhead

**Figure 11.**
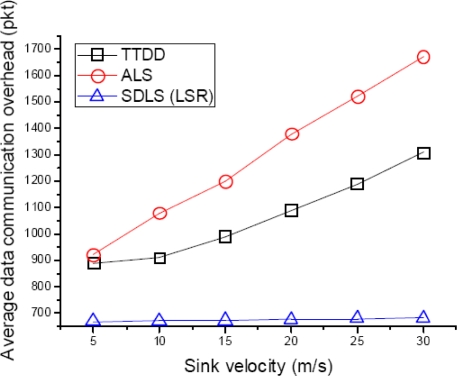
Average data communication overhead.

In order to evaluate the average data communication overhead, we vary the velocity of the sink from 5 m/s to 30 m/s in increments of 5 m/s. Each source node generates a data packet 10 times. In other words, Figure 11 shows that after finishing location service, data packets are sent by multi-hop transmission. We can see that average data communication overhead is the highest with ALS. TTDD consumes less overhead than ALS. This is because TTDD can find a new path by updating to a new grid node. However, this is not the shortest path. Therefore SDLS performance is the best.

## 5. Conclusions

In this paper, we proposed a Sink-oriented Dynamic Location Service (SDLS) approach to handle sink mobility. We have investigated the problem of the mobile sink in WSNs as follows: 1) We provided the global grid structure to solve excessive system overhead due to the grid structures of data dissemination points for each source node. 2) We provided an Eight-Direction Anchor (EDA) system that acts as a location service server to prevent intensive energy consumption at the border sensor nodes. 3) We provided a Location-based Shortest Relay (LSR) to efficiently forward (or relay) data from the source node to the sink with minimal path delay. The experimental result demonstrated that the proposed protocol based on EDA and LSR significantly reduces energy consumption and communication overhead.

## Figures and Tables

**Table 1.** Simulation variables.

Network size	1000 m × 1000 m
α_11_, α_12_	80 nJ/bit
α_2_	100 pJ/bit/m^2^
Packet size (control, data)	36 bytes, 64 bytes
Transmission range	200 m
Distribution type of sensor nodes	uniform
Model of the sink mobility	Random way-point
